# Spatial Distribution of Fine Particulate Matter in Underground Passageways

**DOI:** 10.3390/ijerph15081574

**Published:** 2018-07-25

**Authors:** Xin-Yi Song, Qing-Chang Lu, Zhong-Ren Peng

**Affiliations:** 1 Center for Intelligent Transportation Systems and Unmanned Aerial Systems Applications, State Key Laboratory of Ocean Engineering, School of Naval Architecture, Ocean & Civil Engineering, Shanghai Jiao Tong University, Shanghai 200240, China; songxinyi618@sjtu.edu.cn; 2 China Institute for Urban Governance, Shanghai Jiao Tong University, Shanghai 200240, China; 3 Department of Urban and Regional Planning, University of Florida, Gainesville, FL 32611, USA

**Keywords:** underground passageway, ventilation, PM_2.5_, computational fluid dynamic (CFD)

## Abstract

The unfavorable locations of underground infrastructures and poor ventilation facilities can result in the deterioration of enclosed air quality. Some researchers have studied air quality and ventilation measures in different types of underground buildings. However, few studies have investigated the pollution in pedestrian passageways connecting underground structures. Hence, in this paper, we attempted to investigate the spatial distribution of fine particulate matter (PM_2.5_) in underground passageways. First, measurements were designed and conducted in a pedestrian passageway beneath the Shanghai South Railway Station, Shanghai, China. Second, numerical simulations were performed based on computational fluid dynamic (CFD) technology. Finally, the numerical simulations were extended to examine impacts of the ventilation measures on PM_2.5_ concentration with different inlet positions and air velocity in underground passageways. The simulation results showed good agreement with the experimental data, and the numerical model was validated to be an effective method to investigate the spatial distribution of PM_2.5_ in underground passageways. Results suggest that building additional entrances is an advisable method for improving air quality in the underground passageways of the Shanghai South Railway Station, while jet fans are not recommended. Findings of this study offer suggestions for mitigating PM_2.5_ pollution in underground passageways.

## 1. Introduction

In recent years, a large number of underground structures have been built so as to alleviate the inhospitable conditions of urban land shortage and traffic congestion. According to Xinhua News, in 2015 China became the world’s largest power both in scale and speed of underground construction works [1]. Underground works in road tunnels and urban rail tunnels account for a large part of the underground works in China. By the end of 2016, 30 cities in mainland China began to operate urban rail transit, with a total length of 2564 km of underground lines [2]. In addition, a large number of underground parking lots and shopping malls have been built in Chinese cities. These underground structures offer many advantages, such as superior soundproofing, high earthquake resistance, excellent thermal insulation, and the ability to preserve aboveground landscapes [3]. However, in order to save total costs during design and construction, issues like pedestrian comfort and human exposure to pollutants are often overlooked. Therefore, recently, the air quality problem in underground development has attracted the interest of scholars.

In the literature, researchers have studied air quality in different types of underground structures. El-Fadel et al., conducted measurements of pollutant concentrations in the Salim Slam tunnel [4]. They found that the NO_2_, CO, lead, and beryllium concentrations were several times higher than international standards. Total suspended particulate (TSP) concentration was alarmingly high. Moreno et al. launched an air quality monitoring campaign on differently designed station platforms in the Barcelona subway system [5]. The average concentrations of PM_1_, PM_2.5_, PM_10_ in single-track tunnels were lower than those in double-track tunnels. These levels increased when tunnel ventilation was switched off. Cui et al., verified the presence of metallic iron and oxidized αFe in subway-deposited particles [6], which revealed that the intrinsic nature of rail abrasion was a source of particulate matter in subway tunnels. Zhao et al. measured seasonal patterns of PM_1_, PM_2.5_, and PM_10_ concentrations in a naturally ventilated residential underground garage [7]. Papakonstantinou et al., used computational fluid dynamic (CFD) software to simulate the CO concentration of a typical underground garage in the urban are of Athens, Greece [8]. The study implied that the CO concentration would decrease under appropriate ventilation conditions. Shang et al., found formaldehyde and total volatile organic compounds in underground shopping malls [9]. They also claimed that underground floors had poorer indoor air quality than floors above ground because of the enclosed structure and poor natural ventilation.

For now, studies on air quality and ventilation in underground structure are limited to urban tunnels, subway systems, underground garages, and underground shopping malls. This is due to the high density of people in these places, their close contact with people, and the high concentration and toxicity of the pollutants. In fact, in addition to these underground structures with specific functions, the air quality in the pedestrian passageways connecting underground structures, such as urban underpasses and station transfer corridors, often feature serious pollution problems. The poor air quality in these passageways should also be investigated. The problems originate from the following aspects: (1) Great pollution intensity and wide impact caused by significant numbers of people; (2) Resuspended particle matter due to the absence of adequate frequent floor cleaning; (3) Greater ventilation difficulty caused by a long narrow space structure; and (4) Simple exhaust systems because, given economic considerations, only natural ventilation is used in most underground passageways.

Few studies, heretofore, have been conducted on the air quality of underground passageways designed for pedestrian traffic. After measurement, we have revealed that the main pollutant in underground passageways is particulate matter. Considering that there are few other sources of particle matters in the passageways, pedestrians become the main source, as well as the main victims. Some scholars have concentrated their inquiries on particle resuspension influenced by human activities and people’s exposure to particulate matter; the scope of these studies includes traditional indoor environments such as residences [10], offices [11], and laboratories and rooms constructed for simulations [12,13]. Additionally, studies have confirmed that particle resuspension from flooring due to foot traffic is a source of particulate matter concentration in indoor buildings. Kopperud et al. compared the contribution of outdoor sources with indoor resuspension activities [14]. They found that human activities like walking and vacuuming directly influence indoor particulate matter concentration, by as much as several times the background level. Moreover, Zhang et al., developed particle detachment and resuspension models, using simulations that indicated that multiple factors such as shoe bottom roughness, shoe size, and walking velocity would affect indoor particulate matter concentrations [15]. Ferro et al., revealed the relationship between the strength of the source and the number of people, type of activity, and kind of floor [16]. Their experiment revealed that the average source strength of people walking is approximately 0.21 mg/(min·person). In addition to studies on the sources and distribution of pollutants, it is more practical to explore measures to reduce the concentration of pollutants in underground passageways. The cheapest solution is the use of more intensive cleaning practices. Another method is to strengthen the ventilation, which is more effective. That is to say, cleaning alone is far from sufficient because cleaning practices are unable to remove the particle matter carried by the pedestrians. It is advisable to draw lessons from typical underground structures, since aboveground indoor ventilation systems belong to new systems requiring air conditioning but most underground passageway designers prefer not to utilize air conditioning in order to conserve energy. More precisely, internal ventilation can be improved by the adjustment of the air velocity inside as well as the vent configuration, thus ultimately reducing the concentration of pollutants. Using CFD software, Khalil and Gomaa simulated four scenarios in an underground garage; the results suggested that either too high or too low air velocity would cause problems [17]. A computational analysis of a ventilation system in a subway tunnel was carried out by Kim et al. [18], in which the best vent location on the ventilation performance was chosen from four locations.

For investigating the distribution of walking-induced particles in underground structures, particularly at the physical height of human respiration, an underground passageway beneath the Shanghai South Railway Station was taken as an example. After obtaining the temporal and spatial characteristics of pollutants, the effects of different ventilation schemes on reducing the pollutants were explored. Only the distribution of pollutants was investigated in this paper. Hence, other processes, such as formation and decomposition, were not considered. PM_2.5_ was selected as the object of study because of the uniqueness of this pollution source and the harmfulness to pedestrians. Both field observations and simulations were conducted. Numerical models were built based on physical features calibrated by field monitoring. The CFD software ANSYS FLUENT 15.0 was used to implement numerical simulations. Results of this study can provide advice and references for designers of underground passageways.

## 2. Materials and Methods

### 2.1. Experimental Site Description

Located in the Xuhui District, the Shanghai South Railway Station is the city’s third largest railway station. During the Spring Festival of 2018, the daily passenger flow of Shanghai South Railway Station reached 67,000 people [19]. The Station can be divided into three basic components: the main station, the North Square, and the South Square, covering a total area of 60.32 hectares. The station is referred as a large comprehensive transportation hub which is connected directly with bus stops, taxi stands and roadways. Consequently, there is a complex transfer passageway network on its ground floor level. With respect to foot traffic, through a length of underground passageways, many people transfer between the Shanghai South Railway Station, the Shanghai South Long-Distance Bus Station, and the metro station.

**Figure 1:**
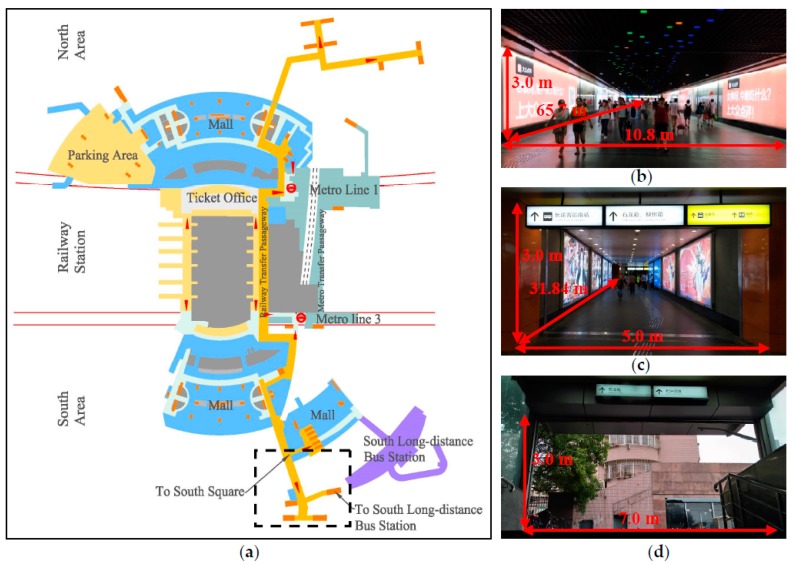
Experimental site in the Shanghai South Railway Station. (**a**) The Shanghai South Railway Station sketch map of the basement floor; (**b**) A view of main segment of the selected passageway; (**c**) A view of the branch of the selected passageway; (**d**) A view of the entrance of the selected passageway.

As shown in Figure 1, the selected passageway is chosen as the experimental site and numerical model prototype for several reasons. Firstly, this passageway connects the Shanghai South Railway Station and the Shanghai South Long-Distance Bus Station. During holidays such as the 2017 Dragon Boat Festival, more than 1200 people entered this passageway each minute, so pedestrians’ exposure to pollutants is more serious than in other passageways with low passenger flow rates. Secondly, except for the passageway with restaurants, which is 42 m away, no other major pollution sources of particulate matter exists, and field measurements proved that pollutants in the passageway bore no relation with the restaurant fumes. Thus, the floating PM_2.5_ in this passageway is considered to be caused mainly by foot traffic. Thirdly, the ventilation conditions in the passageway are relatively poor. Unlike subway stations, the passageways beneath the Shanghai South Railway Station are not equipped with air conditioners. Due to their bending shape and narrowness, outdoor winds do not reach within them. As a result, the weak air renovation makes air inside the passageway turbid; thus, it is imminent to rectify the ventilation system.

### 2.2. Measurements and Field Campaigns

We visited the Shanghai South Railway Station in two separate but related project campaigns during the summer of 2017 and 2018. Field measurements for concentrations of contaminants including PM_2.5_, PM_10_, CO, and volatile organic compounds (VOC), as well as data on meteorology and pedestrian flow were taken in these campaigns. Monitoring periods lasted from 10:00 to 18:00, a time period during which, compared to other periods of the day, pedestrian flow and pollution concentrations are both high. Before each experiment, the Shanghai Environmental Monitoring Center (SEMC) professionally calibrated the relevant instruments. The first campaign was conducted on 1 May (Monday, Labor Day) in several underground structures of the Shanghai South Railway Station, including shopping malls, transfer corridors, metro platforms, and so on. The aim of this campaign was to reveal the ponderance of air pollution in underground passageway by comparing PM_2.5_ concentrations in underground passageway with other types of underground structures. The instruments were fixed on tripods measuring 1.5 m in height and were carried by the experimenters. Finally, the statistics over 8 h in each structure were used in the analysis. The second campaign was conducted on 24 May (Wednesday, a workday), 28 May (Sunday, Dragon Boat Festival), and 1 July (Saturday, a weekend) 2017 and 5 July (Thursday, a workday) 2018 in order to explore air pollution, especially the spatial and temporal distribution of PM_2.5_ in the underground passageway. The specific experimental schemes of the campaigns are introduced as follows.

#### 2.2.1. Pedestrian Data

The scenes of pedestrians walking inside the passageway are manually recorded by cameras during monitoring periods. The shooting positions are set at the exit, the entrances, and the middle of the passageway, respectively. Then, the average pedestrian density, flux, and walking speed are calculated in reference to the videos. The pedestrian-related data are collected to estimate a mean emission rate for PM_2.5_ inlets in numerical models.

#### 2.2.2. Meteorological Data

Hand-held anemometers (NK-5500, Kestrel, Santa Cruz, CA, USA) record the speed and direction of air blown into the four staircase entrances. The resolution of NK-5500 is 0.1 m/s. The time interval is 2 s. Significantly, the mean wind speed hovers at a lower level, about 0.2 m/s, and it was found that wind directions carried small angles of deflection along the axes of staircase entrances. In order to reduce the complexity of the numerical model, it is assumed that all wind directions are parallel to their axes. Temperature and relative humidity (RH) are recorded by a thermohygrograph (HOBO U14-002, Onset, Bourne, MA, USA). Its time interval is 1 s. The temperature accuracy of HOBO U14-002 is *±*0.2 °C. *The accuracy of RH is ±2.5%. The measurement positions of temperature and RH are the same as those of PM_2.5_, as shown in Table 1.*

#### 2.2.3. Contaminant Sampling

**Figure 2:**
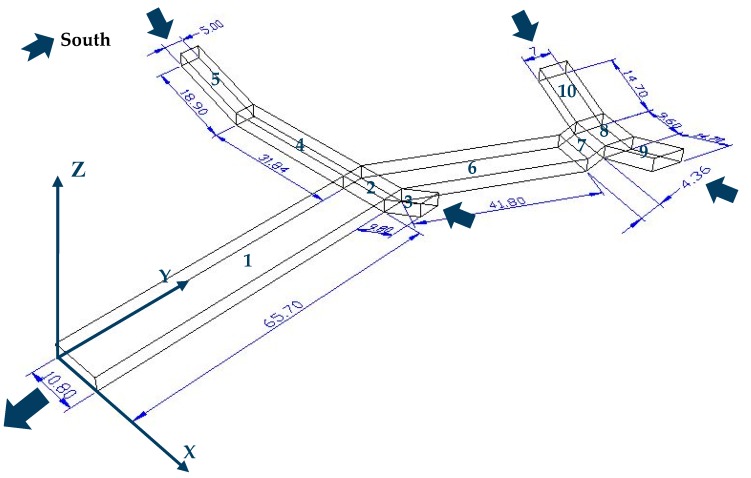
The structure of the selected passageway and the coordinate system. Each segment was assigned a label. Wind directions were marked with arrowheads.

Individual exposure dust meters (SidePak AM520, TSI, Shoreview, MI, USA) measured PM_2.5_ concentrations on 1 May (Monday, Labor Day), 24 May (Wednesday, a workday), 28 May (Sunday, Dragon Boat Festival) and 1 July (Saturday, a weekend), 2017. Their detection sensitivity was 0.001 mg/m^3^. The time interval was 1 s. Aerosol monitors (DustTrak DRX Model 8534, TSI, Shoreview, MI, USA) measured PM_2.5_ and PM_10_ concentrations on 5 July (Thursday, a workday) 2018. Its detection sensitivity was 0.001 mg/m^3^. The time interval was 1 s. An enhanced CO measurer (Model T15x, Langan, San Francisco, CA, USA) was used to measure CO concentration. Its detection sensitivity was 0.01 ppm. The time interval was 1 s. A hand-held volatile organic compounds (VOC) gas detector (PGM-7340, RAE, San Jose, CA, USA) was used to measure VOC concentration. Its detection sensitivity was 1 ppb. The time interval was 1 s. In order to investigate the impacts of pollution concentration on humans, the instruments were mounted on tripods for this measurement, assuming the average human head, upright, stands 1.5 m high [20]. The tripods were carried by experimenters and moved uniformly on 1 May 2017 (Monday, Labor Day) and 5 July 2018 (Thursday, a workday) in order to reveal the general situation of PM_2.5_ in several underground structures and contaminants in the underground passageway. The instruments were put in a few special locations in turn on 24 May (Wednesday, a workday), 28 May (Sunday, Dragon Boat Festival), and 1 July (Saturday, a weekend) 2017. The specific horizontal locations are illustrated in Table 1, and the coordinate system in Figure 2 can be used as a reference. Background PM_2.5_ concentrations during the monitoring periods were measured aboveground, outside the four staircase entrances. During calculation, the average value of these four positions was applied.

An additional experiment expected to prove the mutual exclusivity between the fumes produced by restaurants and pollution concentration in the passageway was implemented on 1 July 2017 (Saturday, a weekend). Results show that PM_2.5_ concentration maintained a high value within a 10-m radius of the fume emission sources. Then, the value of the PM_2.5_ concentration in the air presented a sharp reduction with the distance increases, falling to the average value of the entire passageway network. The air in this passageway should be fume-free, as it is more than 40 m away from the outlets.

### 2.3. Numerical Model

#### 2.3.1. Turbulence Model and Discrete Phase Model

All the simulations in this study were performed using the ANSYS FLUENT 15.0 software program (ANSYS, Inc., Canonsburg, PA, USA). A simple scheme of pressure-velocity coupling is applied. The calculation is based on the standard *κ*-*ε* model (two equations). Previous studies have validated the effectiveness of this model in indoor air flow [21,22,23]. Although there are some limitations with the model, the simulation results are able to provide reasonable accuracy in comparison with field measurement. The standard *κ*-*ε* turbulence model is suitable for simulating airflows and turbulence in semi-closed buildings, such as rooms with windows and underground passageways. The *κ*-*ε* equation is described below [24]:

(1) $$\frac{\partial }{\partial t}(\rho \kappa )+\frac{\partial }{\partial x_{i}}(\rho \kappa \mu_{i})=\frac{\partial }{\partial x_{j}}\left[\left(\mu +\frac{\mu_{t}}{\sigma_{k}}\right)\frac{\partial \kappa }{\partial x_{j}}\right]+G_{\kappa }+G_{b}-\rho \varepsilon \;$$

(2) $$\frac{\partial }{\partial t}(\rho \varepsilon )+\frac{\partial }{\partial x_{i}}(\rho \varepsilon \mu_{i})=\frac{\partial }{\partial x_{j}}[(\mu +\frac{\mu_{t}}{\sigma_{\varepsilon }})\frac{\partial \varepsilon }{\partial x_{j}}]+C_{1\varepsilon }\frac{\varepsilon }{\kappa }(G_{\kappa }+C_{3\varepsilon }G_{b})-C_{2\varepsilon }\rho \frac{\varepsilon^{2}}{\kappa }\;$$

Here, *κ* is defined as kinetic energy of turbulence flow; *ε* as the rate of turbulent dissipation transport; *ρ* is the density; *t* is time; *x_i_* and *x_j_* represent the Cartesian coordinate system; *μ_i_* is the velocity of flow; *μ* is kinetic viscosity; *G_κ_* is turbulence production item; *G_b_* is the turbulent kinetic energy generation caused by Buoyancy; *σ_κ_* and *σ_ε_* are the turbulence coefficients, *σ_κ_* = 1.0, *σ_ε_* = 1.3; $\mu_{t}=\rho C_{\mu }\frac{k^{2}}{\varepsilon }$, *C_μ_* = 0.09; *C*_1*ε*_ = 1.44, *C*_2*ε*_ = 1.92, and *C*_3*ε*_ = 1.44.

As there are comparatively fewer particles in the air, a discrete phase model was used. For the aerosol movement near the ground, the Euler method was used. The trajectory of particles in the passageway was tracked by Lagrange’s method and simulated by the random walk model in the random track model. The effects of diverse forces such as Saffman, drag, and gravity can be assessed. The dynamic equations of particulate transport can be expressed as follows [25]:

(3) $$\frac{\pi d_{p}^{3}\rho_{P}}{6}\frac{du_{P}}{dt}=F_{drag}+F_{gravity}+F_{saffman}\;$$

(4) $$F_{drag}=-\frac{1}{6}\pi d_{P}^{3}\rho_{P}\frac{1}{\tau }(u_{P}-u)\;$$

(5) $$F_{gravity}=\frac{1}{6}\pi d_{P}^{3}(\rho_{P}-\rho )g_{i}\delta_{i}\;$$

(6) $$F_{saffman}=\frac{1}{6}\pi d_{P}^{3}\rho_{P}\frac{5.188v^{0.5}d_{ij}}{Sd_{P}{\left(d_{lk}-d_{kl}\right)}^{0.25}}(u_{P}-u)\;$$

where *u_P_* is the particle velocity; *u* is the fluid velocity; *ρ_P_* is the particle density; *ρ* is the fluid density; *d_P_* is the particle diameter; *S* is the density ratio between a particle and adjacent fluid; *ν* is the kinematic viscosity; *δ_i_* is the unit delta function; *g_i_* is the hydrodynamic viscosity; *τ* is the particle relaxation time; and *d_ij_* = (*u_ij_* + *u_ji_*)/2 is the deformation rate tensor.

#### 2.3.2. Computational Domain and Mesh Discretization

The geometry of computational domain is set to the inner space of the underground passageway. Pedestrian models are put inside the domain as cylinders with fixed intervals and quantities. To focus on the research issues, the entire passageway was abstracted into a hollow geometry, ignoring the interior lights, billboards, guide signs, drains, and other material. Specific dimensions are listed in the previous section. Pedestrians were constructed into upright cylinders. According to the actual size of the human body, the bottom of each cylinder was set as an ellipse with a long axis of 0.50 m and a short axis of 0.20 m. The height of cylinders was set as 1.70 m.

**Figure 3:**
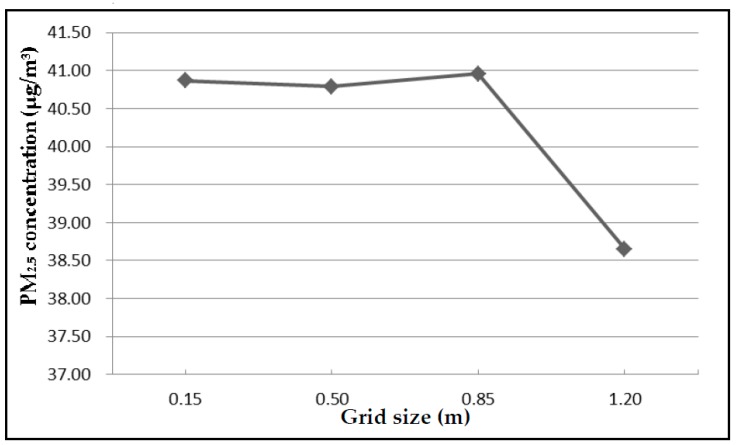
The relationship between grid size and average PM_2.5_ value at the height of 1.5 m.

**Figure 4:**
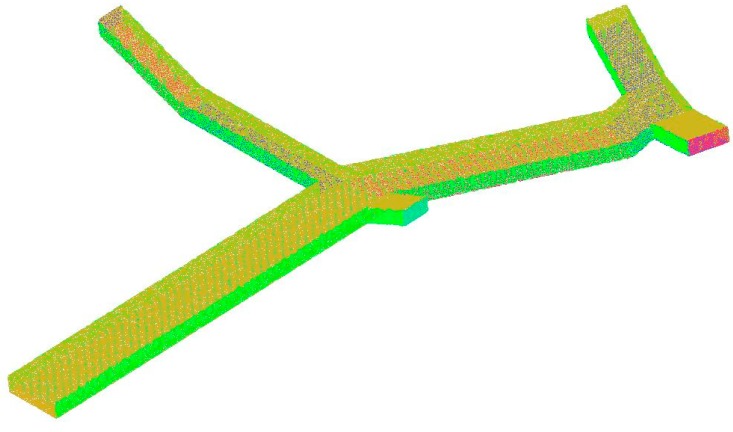
The mesh discretization of the computational domain.

Meshes in the computational domain were divided in ICEM CFD 15.0. It’s an integrated computer engineering and manufacturing software for computational fluid dynamics. In this case, three layers of prism meshes were applied to cover pedestrian surfaces, with a maximum element size of 0.1 m, increasing by a growth factor of 1.2. Pedestrians were designed as impenetrable entities, so there was no need to partition meshes inside the cylinders. The remaining space in the passageway was filled with grids with a maximum element size of 0.5 m. Grid independency was checked after mesh discretization. As shown in Figure 3, the average PM_2.5_ at the height of 1.5 m remained steady until reaching a grid size of 0.85 m; then, an abnormal fluctuation appeared. Hence, it was reasonable to choose 0.5 m as the grid size. Finally, grid independency was achieved with a 3D mesh of 5,172,334 unstructured tetrahedral nodes. Besides, standard wall functions were used for near-wall treatment. The mesh discretization of the computational domain is shown in Figure 4.

Later on in the experiment, as a measure of enhanced ventilation, a jet fan (an object widely employed in highway tunnels’ ventilation systems) was introduced into the model. Generally, jet fans are hung on the tops of both sides of a tunnel; they do not occupy traffic areas. The jet fan used in this study was SDS(R)-4.0-2P-4-18° [26], a device with a performance similar to the jet fan (Axalu TR 40 RCS) used by Khalil and Gomaa [17]. The jet fan was simplified into a cylinder, with a length of 2.03 m and a diameter of 0.40 m. Similar to pedestrian simulations, the fan had no meshes inside and was covered with three layers of prismatic grids outside.

#### 2.3.3. Boundary Conditions

Boundary conditions are determined by an analysis of the model and calculation of relevant parameters, which are shown in Table 2.

As noted in previous studies, emission sources are always replaced by line sources on a roadway [27,28], and this practice serves as a point of reference for this study, when a plane source is paced on the floor of the passageway. These two surfaces measure the same size, but the difference is that here the plane source is dug out of areas equal to the bottom of the cylinder. This practice avoids the potential effects of surfaces overlapping on the pollutant injection. Additionally, because field observation shows there is no obvious aggregation of pedestrians in the passageway and that distances between pedestrians are relatively even, it is supposed that the emission of the plane source is uniform.

All numerical simulations are based on 28 May (Sunday, Dragon Boat Festival) from 10:00 to 11:00 in the experimental scenario, which was the time period with the highest PM_2.5_ concentration. In order to investigate the PM_2.5_ generated within the passageway, the air blowing from the four entrances was set to contain no PM_2.5_. The emission rate was estimated based on previous research which demonstrates the average source strength of people walking was 0.21 mg/(min∙person) [16], and measured visitor flow during monitoring periods. According to the field videos, distances between pedestrians were very short that day, especially in segments 1, 2, and 6. Then, the number of pedestrian models fixed in the passageway was determined by these separation distance. Finally, about 379 pedestrian models are set in the passageway and the total emission rate of floor is 1326.5 μg/s. The emission rate of each segment is related to number of pedestrians on its floor.

There are several different versions of injection speed applied. Eisner et al., employed particle imaging velocimetry (PIV) to capture the major characteristics of airflow generated by the human foot in motion [29]. They concluded that particles are detached from the foot or floor to become airborne due to the airflow produced by the foot’s rotation. The average vertical velocity was about 5 cm/s. However, Zhang et al., claimed that a small cloud of resuspension particles with a vertical expansion radius of 17 cm/step form with every step [15]. In this model, a compromise is adopted and the vertical injection rate was set as 10 cm/s. After all, this study focuses on the distribution of PM_2.5_ under adverse ventilation conditions, rather than the impacts of foot traffic on PM_2.5_ diffusion. Thus, the pedestrians were set as fixed for simplicity. All particulate matter was ejected along the normal direction of the floor.

In order to simplify calculation, the jet fan’s nozzle velocity was presupposed to be uniform. The front and back ends of the jet fan were regarded as two velocity inlets with air velocity of equal magnitudes. The air velocity was determined according to the actual specifications of the jet fan. The outer walls of the fan were assumed to be adiabatic and qualified as walls with no-slip velocity boundary conditions.

## 3. Results

### 3.1. Field Measurements

#### 3.1.1. PM_2.5_ Concentration in Different Locations

Several possible contaminants were monitored in the underground passageway on 5 July 2018. As shown in Table 3, the concentration of CO in underground passageway is very low, and as such might not be harmful to pedestrians. The concentration of VOC in underground passageway is not high compared with that of shopping malls, with the average VOC concentration of equaling 176 ppb. This is due to the large number of wares and luxurious decoration in the underground shopping malls. In contrast, the concentration of particulate matter inside the underground passageway should be paid more attention. The result of correlation analysis shows that PM_2.5_ and PM_10_ are correlated significantly (r = 0.926, *p* < 0.01, two-tailed), suggesting that both types of matter are derived from the same sources. Besides, the concentration of PM_2.5_ accounts for about 74% of the PM_10_ concentration in the underground passageway. PM_2.5_ was chosen for analysis as it is more harmful to humans than PM_10_.

The underground network under the Shanghai South Railway Station expands as a multifarious layout of shopping malls, open-air areas, subway stations, a transfer route between line 1 and line 3, and so on. As a point of contrast, PM_2.5_ levels at these various sites are also monitored. Table 4 shows the minimum, maximum, and average PM_2.5_ concentrations along with the standard deviation for the ten measurement sites from 10:00 to 18:00 on 1 May 2017. As an international holiday, that is, the Labor Day, the flowrate of visitors of this day remained high and stable.

As shown in Table 4, shopping malls have the lowest average concentration of PM_2.5_. Because these large stores keep their spaces air-conditioned all day long, the fresh air circulation system could effectively reduce the PM_2.5_ concentration and make the air quality better than in the South Square. The square is a wide, open-air, green environment, with a spacious terrain and reduced amount of pedestrian traffic, resulting in a low and stable PM_2.5_ concentration. In addition to the South Square, another short section of open area, below ground level, connects the passageways that stretches from under the South Square to the main station. Although they both are exposed to the external air, due to a lower position and the surrounding buildings that impeded the natural flow of air, the PM_2.5_ concentration of the open-air section is significantly higher. The same phenomenon appears on the metro platforms. As the Shanghai South Railway Station is the transfer station of Metro line 1 and line 3, the entrances of the two lines are located on either of the station’s sides and are connected via a transfer corridor. Despite the positional symmetry, the platform of line 3 is above ground and directly exposed to ambient air, while the platform of line 1 is situated on the second floor of the underground. In this environment, once PM is blown to the platform when the train passing by, it cannot not be diluted, leading to a 24% increase in average PM concentration. Such a significant contrast illustrates the importance of efficient ventilation.

When comparing the metro transfer corridor with railway transfer corridor, the data on the pedestrian passageway leads us to a similar conclusion. The pedestrian passageway in the south refers to the corridor connecting the Shanghai South Railway Station with Shanghai South Long-Distance Bus Station. More than 1000 people entered this passageway every minute during the observation period on 1 May (Monday, Labor Day). In the station’s entire underground network, these three corridors are the busiest sections, and the differences in pollution concentration are correlated directly with ventilation conditions. The Shanghai Metro utilizes independent ventilation devices, so PM_2.5_ concentration in the metro transfer corridor remains at the lowest level. There is no specific ventilation equipment in the railway transfer corridor, but the openings at both ends and spacious interiors provide plenty of natural wind. Pedestrians in this corridor can feel strong drafts, as the air quality in the railway transfer corridor is good. The ventilation condition of the pedestrian passageway in the south is the poorest. The lack of equipment together with the narrow, zigzagging structure perpetuates this inferior air circulation. The pedestrian passageway in the north is a section structurally similar to the pedestrian passageway in the south. They occupy symmetrical positions in the station and possess a long narrow structure. A key difference in air quality lies, of course, in the rate of passenger flow. Few pedestrians in pedestrian passageway in the north cause less pollution, indicating that pollution sources in passageways are closely related to pedestrian volume.

The particulate matter levels near small restaurants are also monitored. The concentration depends on the cooking method and restaurant types. The grill house produced the most fumes, while the monitoring values outside the bakery do not fluctuate. Therefore, the standard deviation is the greatest in the restaurant areas. Meanwhile, the greatest mean value appears in the pedestrian passageway in the south. This shows that the PM_2.5_ pollution there requires more attention.

#### 3.1.2. Temporal and Spatial Characteristics of PM_2.5_ Concentration

**Figure 5:**
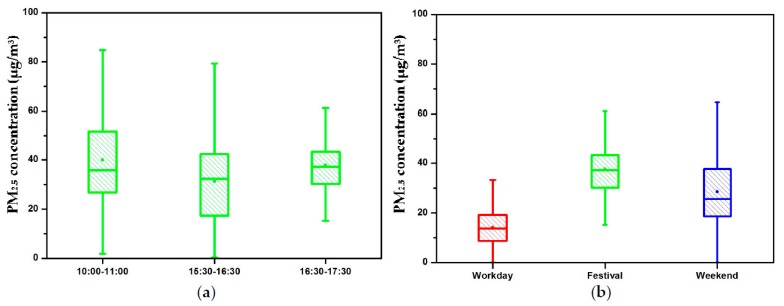
Box plots of PM_2.5_ concentration in the passageway. (**a**) During different time period in a day; (**b**) During different dates.

Figure 5 shows the box plots of PM_2.5_ concentrations measured in the passageway. From then on, the passageway refers to the pedestrian passageway chosen as the experimental site. The background PM_2.5_ concentrations on 24 May (Wednesday, a workday), 28 May (Sunday, Dragon Boat Festival), and 1 July (Saturday, a weekend) 2017 were 21 μg/m^3^, 34 μg/m^3^, and 83 μg/m^3^, respectively. In order to clearly define the differences in PM_2.5_ concentrations produced by pedestrians, background concentrations were removed. Figure 5a displays PM_2.5_ concentrations recorded between 10:00 and 11:00, 15:30 and 16:30, and 16:30 and 17:30 on 28 May (Sunday, Dragon Boat Festival). The 28 May box plot in Figure 5b is marked in green as well, while the red and blue boxes represent the measurements taken on 24 May (Wednesday, a workday) and 1 July (Saturday, a weekend). The experimental data are analyzed by a one-way ANOVA and post hoc test. The results show significance difference between different periods of the day, especially between 10:00 and 11:00 and 15:30 and 16:30, with *p* < 0.05, and 15:30 and 16:30 and 16:30 and 17:30 with *p* < 0.05. A significant difference among different dates appeared as well, all with values of *p* < 0.05. The smaller difference between 10:00 and 11:00 and 16:30 and 17:30 might be because the time periods correspond to similar passenger flow patterns. During the Dragon Boat Festival on 28 May, there were many people leaving the city in the morning and returning at nightfall. The average values of walking-induced PM_2.5_ concentrations of these three periods were 40 μg/m^3^, 31 μg/m^3^, and 38 μg/m^3^. This means that, due to the fluctuation of passenger flow, the percentage difference in PM_2.5_ concentration within a day could reach up to 30%. The same difference is reflected in the comparison of dates. The red, green and blue boxes in Figure 5b represent a workday, festival day, and weekend day, respectively. The measurement period was from 16:30 to 17:30, a rush hour period for passengers returning to the city. The average PM_2.5_ concentrations of this time period of these days were 14 μg/m^3^, 38 μg/m^3^, and 28 μg/m^3^. Again, a significant differences derive from passenger flow rates, as they are often significantly higher on holidays than normal weekdays. Compared to 24 May (Wednesday, workday), on 28 May (Sunday, Dragon Boat Festival) a 171% increase occurred in the average PM_2.5_ concentration. As mentioned above, background concentrations were removed in the analysis.

**Figure 6:**
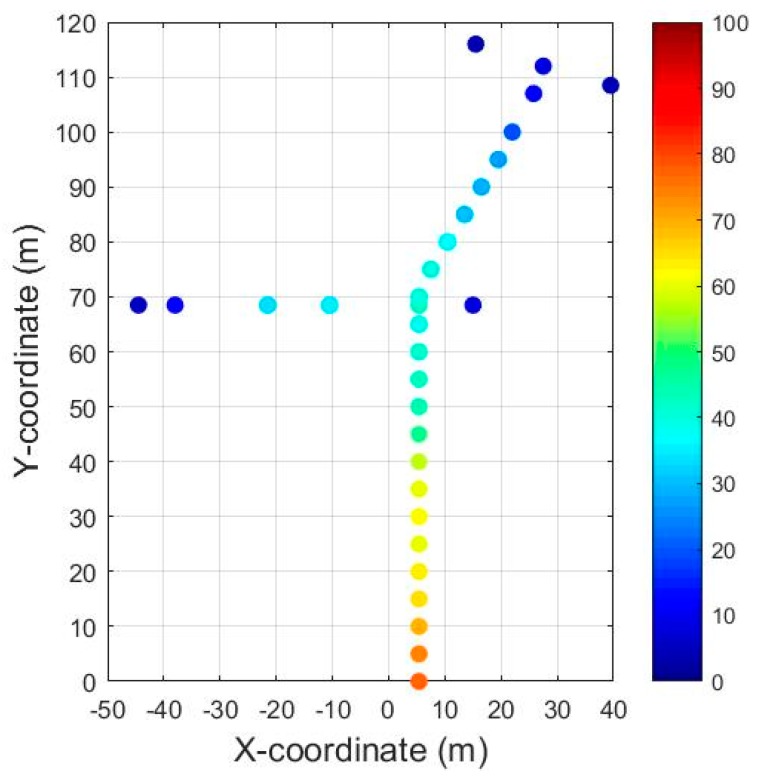
Scatter plots of PM_2.5_ concentration at sampling sites in the passageway on 28 May (Sunday, Dragon Boat Festival).

Figure 6 shows the scatter plots of average PM_2.5_ concentration measured at the sampling sites in the passageway on 28 May (Sunday, Dragon Boat Festival). Background concentration was still removed this time. The varying tendency along the passageway is presented clearly. The highest level of PM_2.5_ concentration appears in segment 1 (the labels of segments are displayed in Figure 2). The concentration in segments near the entrances are low except for in segment 2, which is a connection of three segments with high-density pedestrian flow and also a gathering place for PM_2.5_ from the three segments. The results show that the spatial distribution of PM_2.5_ are affected by the pedestrian flow and ventilation.

### 3.2. Validation of the Numerical Model with Field Data

**Figure 7:**
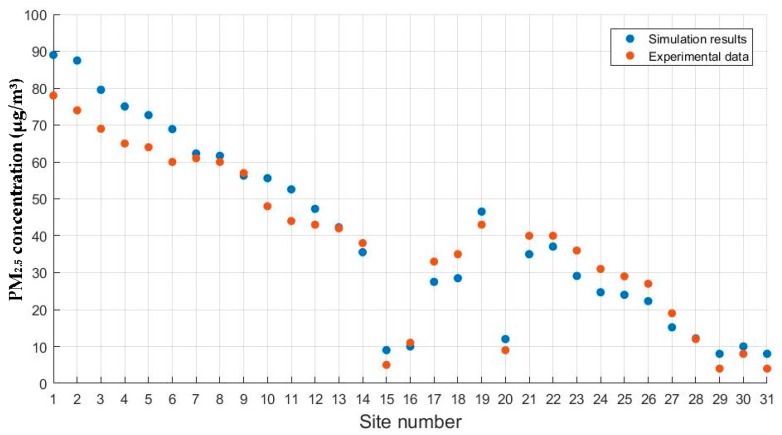
Scatter plots of PM_2.5_ concentration at sampling sites.

Results obtained from numerical simulations were evaluated by field observations for effectiveness of parameters used in numerical models. An agreement of distribution patterns of PM_2.5_ concentrations between numerical simulations and field observations is highlighted. Similarly, we took the PM_2.5_ concentrations at the height of 1.5 m measured at the 31 sampling sites from 10:00 to 11:00 on 28 May (Sunday, Dragon Boat Festival) as examples. As shown in Figure 7, the distribution variation tendency of PM_2.5_ concentrations obtained from numerical simulations shows a relatively good agreement with field observations. In the present article, the validation measure also consists of a statistical procedure by means of Pearson correlation. The results show that they are highly correlated with the correlation coefficient equaling 0.979, and the *p* value is smaller than 0.01. At this point and by means of these results, we validated the numerical model with field data.

### 3.3. Effects of Ventilation Mode

**Figure 8:**
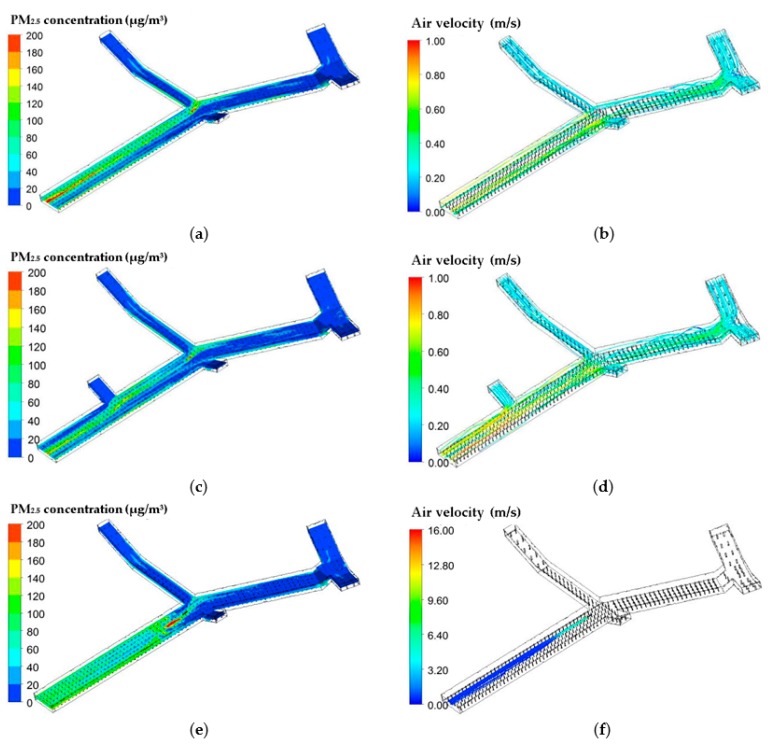
PM_2.5_ distribution at the height of 1.5 m and wind streamline of the passageway with different ventilation modes. (**a**) PM_2.5_ concentration at the height of 1.5 m in the passageway without ventilation equipment; (**b**) Wind streamline in the passageway without ventilation equipment; (**c**) PM_2.5_ concentration at the height of 1.5 m in the passageway with an additional entrance; (**d**) Wind streamline in the passageway with an additional entrance; (**e**) PM_2.5_ concentration at the height of 1.5 m in the passageway with a jet fan; (**f**) Wind streamline in the passageway with a jet fan.

The air quality of segment 1 was the poorest, and may need the most ventilation modification. Therefore, three scenes were simulated and corresponding effects were contrasted. First, the additional entrance was built at the midsection of segment 1. Subsequently, all entrances worked together to improve the ventilation condition. As shown in Figure 8d, the wind streamlines in segment 1 increase significantly, and the average PM_2.5_ concentration, at the height of 1.5 m which is assumed to be the breathing height of humans, is lowered to 48 μg/m^3^ with a reduction of 21%.

Although the jet fan is a common ventilation device employed in tunnels and underground garages, its effect on air quality in the underground passageway is far from satisfactory. As shown in Figure 8f, the wind released from the fan has a high velocity and runs through the entire segment. However, the average PM_2.5_ concentration, at the height of 1.5 m in segment 1, does not decrease; but increases to 72 μg/m^3^ instead. This phenomenon could be explained by the vertical distribution of PM_2.5_ in segment 1. In the case of natural ventilation, as the height is reduced from 3 m to 1.5 m, the PM_2.5_ concentration shows a gentle downward trend. On the contrary, when a jet fan is installed, PM_2.5_ concentrations at the heights of 3 m, 2.5 m, 2 m, and 1.5 m, are 21 μg/m^3^, 29 μg/m^3^, 60 μg/m^3^, and 72 μg/m^3^. The horizontal air velocity above the heads of pedestrians is so high that the PM_2.5_ cannot not spread to a higher level of altitude. Moreover, in order to remove the accumulation of pollutants, the air velocity of the jet fan is higher. From a perspective of public safety, the strong wind in the passageway could cause pedestrian discomfort and might be harmful. Therefore, to modify underground passageways, natural ventilation, rather than jet fans, should be applied.

### 3.4. Effects of Inlet Air Velocity

In order to reveal the distribution of PM_2.5_ at breathing height in the passageway, a specific calculation was carried out. First, a horizontal plane with a height of 1.5 m was intercepted in the passageway. Then the whole plane was divided into several small planes with a width of 5 m. Afterwards, area average of PM_2.5_ concentrations of each small plane are computed. The calculation is equivalent to exploring the distribution of PM_2.5_ along the passageway. This method was also applied to the following sections. Analysis is carried by varying the magnitudes of the air velocity entering the four staircase entrances. In this section, no extra entrances are built. The objective was to investigate the influence of inlet air velocity on ventilation effect. The direction of wind and coordinate axis is shown in Figure 2.

**Figure 9:**
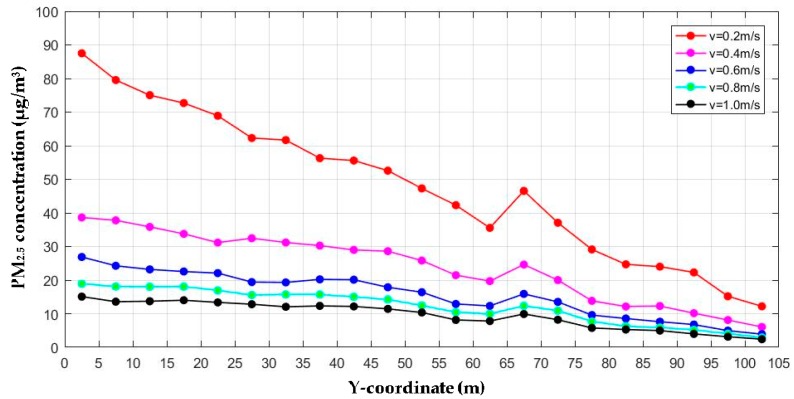
PM_2.5_ concentration profiles for different inlet air velocity.

The curves in Figure 9 present a very obvious downward trend, except for an upward turning point in the middle of 65 m and 70 m. This is in good agreement with the filed measurements. The area between 65 m and 70 m is the joint of two long passageway sections. The pollutants from the two passageway sections are gathered here, forming a small peak of PM_2.5_ concentration. The area of 0 to 65 m is the most densely populated section, and it is no wonder that the PM_2.5_ concentration in it is the highest. The entire passageway ends with two symmetrical entrances at 105 m. Consequently, the closer to the end of passageway, the lower the PM_2.5_ concentration.

All the five curves have a similar tendency, but the specific values are different. With the increase in inlet air velocity, the PM_2.5_ concentration in the whole passageway decreases. There is a leap in the ventilation effect when the inlet air velocity increases from 0.2 m/s to 0.4 m/s. However, as the inlet air velocity continues to increase, the improvement of ventilation diminishes significantly. When the inlet air velocity increases to 0.8 m/s, the PM_2.5_ concentration of the whole passageway is controlled under 20 μg/m^3^, which is quite low. There is no need to increase the inlet air velocity to reduce pollution. Even if the inlet air velocity increases to 1.0 m/s, the decrease in PM_2.5_ concentration is limited. Moreover, we obtain statistically significant linear regressions for the relationship between the *Y*-coordinate and PM_2.5_ concentrations:

- *v* = 0.2 m/s: C_PM2.5_ = −0.702y + 84.894 (R^2^ =0.981, *p* < 0.01).
- *v* = 0.4 m/s: C_PM2.5_ = −0.323y + 40.903 (R^2^ =0.960, *p* < 0.01).
- *v* = 0.6 m/s: C_PM2.5_ = −0.215y + 26.901 (R^2^ =0.958, *p* < 0.01).
- *v* = 0.8 m/s: C_PM2.5_ = −0.161y + 20.574 (R^2^ =0.953, *p* < 0.01).
- *v* = 1.0 m/s: C_PM2.5_ = −0.123y + 16.097 (R^2^ =0.942, *p* < 0.01).

This finding is of practical significance. The underground passageway should be located somewhere with enough natural wind. Meanwhile, it is not necessary to reduce the pollution by selecting the address in a place where the inlet air velocity is too high. A balance can be achieved between the ventilation effect and the cost.

### 3.5. Effects of Entrance Position

From y-PM_2.5_ concentration data for different cases the PM_2.5_ concentration profiles are drawn. The present paper provides eight cases, including the basic scenario with no additional entrance. In these cases, the inlet air velocity was set to a fixed value of 0.2 m/s, which is the mean of measured values in field experiments. Here, y represents the position of the additional entrance in the axis of the underground passageway. The width of the vent was set to be 5 m.

**Figure 10:**
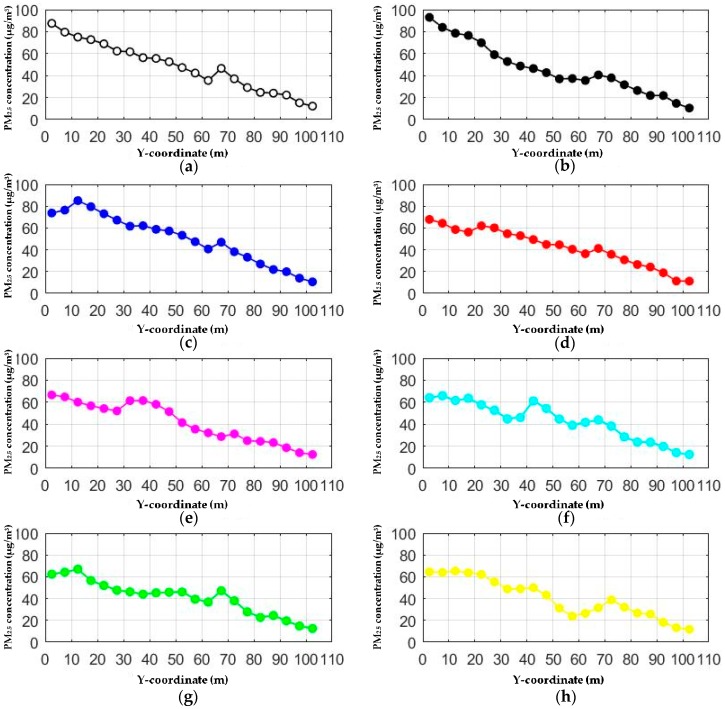
PM_2.5_ concentration profiles for different additional entrance locations. (**a**) Basic scenario with no additional entrance; (**b**) Additional entrance at y = 0 m; (**c**) Additional entrance at y = 10 m; (**d**) Additional entrance at y = 20 m; (**e**) Additional entrance at y = 30 m; (**f**) Additional entrance at y = 40 m; (**g**) Additional entrance at y = 50 m; (**h**) Additional entrance at y = 60 m.

Since the area of 0 to 65 m is the most densely populated, the additional entrance was only set in this area in order to make the ventilation improvement more pertinent. Figure 10 shows that the area of 65 to 105 m is almost unaffected. This indicates that entrances should be set up for each section of a long passageway. In general, ventilation improvements in most cases are effective. The second curve and the third curve have anomalous properties. The PM_2.5_ concentration at the beginning of passageway in Figure 10b is higher than that of the basic scenario. The PM_2.5_ concentration in Figure 10c is almost higher than that of the basic scenario at all times. This phenomenon shows that vents should not be built at backward positions. The wind that comes from along the entrance will hamper the dilution of the air.

Another special phenomenon is the sudden increase in the PM_2.5_ concentration at the additional entrance position, which can also be found in Figure 9. The cause of this phenomenon is consistent with the jet fan. The air blowing into the entrance causes the air velocity above people’s heads to speed up, but the pollutants are hindered by the existence of the pedestrians’ bodies under 1.5 m. The increase in the concentration at the height of 1.5 m does not represent an increase in the overall concentration. All of the average PM_2.5_ concentrations of Section 1 decline in these cases except for the case in which y = 10 m. The reduction is of at least 14%.

## 4. Discussion

This paper draws attention to the spatial distribution of fine particulate matter in underground passageways. Measurements were taken under the Shanghai South Railway Station. Then, the average concentration was used for comparisons and analysis. Average values make data simple and measurable, but such rough analysis may lead to information loss. For example, by comparing average PM_2.5_ concentration in different kinds of underground structures, we concluded that the better the ventilation condition is, the lower the pollutant concentration is. In future analysis, we would like to record the whole time series of PM_2.5_ concentration and air velocity in these underground buildings. Then the variation of the PM_2.5_ concentration over time and the direct effect of air velocity on PM_2.5_ concentration can be revealed.

A 3D model was built based on the experimental data. Pedestrians in the passageway were simplified as upright cylinders. This assumption ignores the influence of human movement on the distribution of particle matters. In future research, moving mesh can be used to simulate moving pedestrians. This can make the simulation results more consistent with the actual situation.

**Figure 11:**
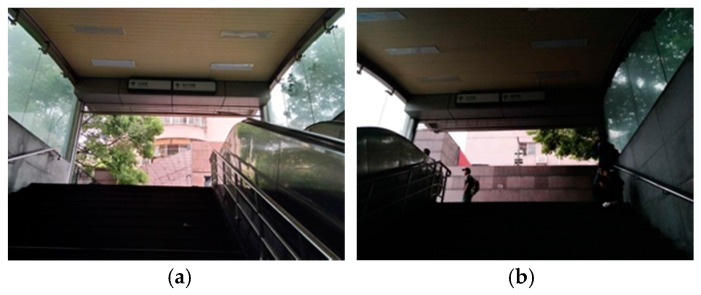
Entrances blocked by the wall. (**a**) The entrance in the southwest end of the underground passageway of Shanghai Railway Station; (**b**) The entrance in the southeast end of the underground passageway of Shanghai Railway Station.

The simulation results show that the pollutant concentration can be reduced by increasing the air velocity at the entrance. However, the increase in inlet velocity may bring secondary side effects such as increasing the suspended PM_2.5_. In this paper, we removed the background concentration in simulation because the PM_2.5_ concentration outdoor is much lower than underground according to our field measurement. The background concentration was also removed in the data analysis process that aimed to explore the PM_2.5_ concentration in underground passageway during different dates and time periods. In future research, the influence of outdoor pollutant concentration on the indoor environment should be fully considered. As shown in Figure 11, barriers are often placed at the entrances of underground passageways, such as walls. In practice, the removal of these walls will increase the inlet wind speed to a certain extent.

## 5. Conclusions

Field measurements of air quality were conducted in underground structures of Shanghai South Railway Station. An underground passageway was chosen as the main research object. Then, numerical calculations were made. The following conclusions were drawn:

(1) Although there are contaminants such as CO and VOC in the underground passageway, particle matter is the main pollutant. There is a strong correlation between PM_2.5_ and PM_10_, indicating their homology.
(2) According to the comparison of pollutant concentration in different locations, it was found that ventilation is a major factor affecting the concentration of pollutants, in addition to the impact of pollution sources.
(3) By exploring the temporal variation of PM_2.5_ concentration in the underground passageway, it was determined that the number of pedestrians also affects the concentration of pollutants. This also confirms that pedestrians are the main source of PM_2.5_ in underground passageways.
(4) Several scenarios with additional entrance were built. Then, numerical analysis was done by changing the inlet air velocity. The results show that the concentration of PM_2.5_ in the underground passageway decreases with the increase of inlet wind speed. Therefore, in practice, it is possible to increase the intake volume and reduce the pollution in the underground passageway by keeping the entrance unblocked and having the entrance in an open area.
(5) Attempts were made to find the optimal position of additional entrance by changing its position in simulation. Results show that vents should not be built at backward positions, and should be built for each section of the zigzag underground passageway.

## Figures and Tables

**Table 1:** PM_2.5_ sampling sites.

Site Number ^1^	*X*-Coordinate ( m)	*Y*-Coordinate (m)
1	5.4	0
2	5.4	5.0
3	5.4	10.0
4	5.4	15.0
5	5.4	20.0
6	5.4	25.0
7	5.4	30.0
8	5.4	35.0
9	5.4	40.0
10	5.4	45.0
11	5.4	50.0
12	5.4	55.0
13	5.4	60.0
14	5.4	65.0
15–20	−44.5, −38.0, −21.5, −10.5, 5.4, 15.0	68.5
21	5.4	70.0
22	7.5	75.0
23	10.5	80.0
24	13.5	85.0
25	16.5	90.0
26	19.5	95.0
27	22.0	100.0
28	25.8	107.0
29	39.5	108.5
30	27.5	112.0
31	15.5	116.0

^1^ The bottoms of each section of the passageway are not on the same horizontal plane, but all measurement sites were located at 1.5 m above the floor.

**Table 2:** Condition types and values of components in the computational domain.

Components	Condition Type	Value
staircase entrances	velocity inlet	*v =* 0.20 m/s
exit	pressure outlet	relative pressure *p =* 0
ceiling	stationary wall	roughness constant = 0.50
wall	stationary wall	roughness constant = 0.50
pedestrian	stationary wall	roughness constant = 0.50
floor-segment 1 and 2	stationary wall	roughness constant = 0.50
	injection source	*v =* 0.10 m/s, total flow rate = 770 μg/s
floor-segment 3	stationary wall	roughness constant = 0.50
	injection source	*v =* 0.10 m/s, flow rate = 21 μg/s
floor-segment 4	stationary wall	roughness constant = 0.50
	injection source	*v =* 0.10 m/s, flow rate = 91 μg/s
floor-segment 5	stationary wall	roughness constant = 0.50
	injection source	*v =* 0.10 m/s, flow rate = 35 μg/s
floor-segment 6	stationary wall	roughness constant = 0.50
	injection source	*v =* 0.10 m/s, flow rate = 322 μg/s
floor-segment 7, 8, 9 and 10	stationary wall	roughness constant = 0.50
	injection source	*v =* 0.10 m/s, total flow rate = 87.5 μg/s
out surface of jet fan	stationary wall	roughness constant = 0.50
inlet and outlet of jet fan	velocity inlet	*v_in_ =* −16.10 m/s, *v_out_ =* 16.10 m/s

**Table 3:** Concentrations of different contaminants in the underground passageway. L.B. is the lower bound of 95% confidence interval; U.B. is the upper bound of 95% confidence interval.

Contaminants	Min.	Max.	Mean.	S.D.	L.B.	U.B
CO (ppm)	2.58	2.62	2.60	0.01	2.59	2.60
VOC (ppb)	76	92	86	4.23	85	87
PM_2.5_ (μg/m^3^)	52	96	71	11.94	69	73
PM_10_ (μg/m^3^)	62	137	96	22.21	92	100

**Table 4:** The minimum, maximum, average PM_2.5_ concentrations, the standard deviation, and confidence interval for sampling locations.

Site	PM_2.5_ (μg/m^3^)					
	Min.	Max.	Mean.	S.D.	L.B.^1^	U.B.^2^
Shopping malls	20	36	26	3.53	25	27
South Square	22	50	37	5.02	36	38
Underground open-air section	31	74	44	7.71	43	46
Metro line 3 platform	28	48	37	4.63	36	38
Metro line 1 platform	34	87	46	9.98	45	49
Metro transfer corridor	35	54	43	4.78	42	44
Railway transfer corridor	34	60	45	6.40	44	46
Pedestrian passageway in the south	58	108	88	11.63	85	90
Pedestrian passageway in the north	30	86	45	8.58	43	48
Small restaurants	31	111	53	14.97	51	56

^1^ L.B. is the lower bound of 95% confidence interval. ^2^ U.B. is the upper bound of 95% confidence interval.
